# The DaNa projects: public communication of (nano)material safety data—from conspiracy theories to study quality

**DOI:** 10.3389/ftox.2024.1382458

**Published:** 2024-05-28

**Authors:** Dana Kühnel, Harald F. Krug, Christoph Steinbach, Katja Nau

**Affiliations:** ^1^ Helmholtz Centre for Environmental Research (UFZ), Department Ecotoxicology (ETOX), Leipzig, Germany; ^2^ NanoCASE GmbH, Engelburg, Switzerland; ^3^ Society for Chemical Engineering and Biotechnology (DECHEMA), Frankfurt am Main, Germany; ^4^ Karlsruhe Institute of Technology (KIT), Institute for Automation and Applied Informatics (IAI), Karlsruhe, Germany

**Keywords:** science communication, safety, nanomaterials, advanced materials, human health, environmental health

## Abstract

In this perspective, the authors give their view on the developments and experiences on communicating on (nano)materials safety. We would like to share our experiences with the scientific community in order to make them useful for future communication activities. We present the long-term work of the science communication projects DaNa, DaNa2.0 and DaNa4.0, running from 2009 to 2023. Starting in the early 2000s with the beginnings of nanotechnology research, communication on the safety of nanomaterials with the public was still very new and faced the projects with many challenges. Today, science communication is indispensable for the dissemination of scientific findings and a fact-based approach like the DaNa “Knowledge Base Materials” creates a trustworthy dialogue with the public. This long-term project series has made a significant contribution to communication on the safety of nanomaterials, perhaps even the largest among publicly funded project series worldwide.

## Graphical Abstract

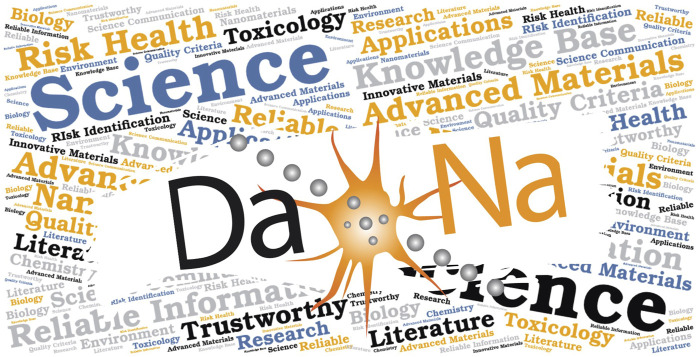

## 1 The projects

There is constant innovation in the (nano)material sector, with a multitude of new materials and opportunities for their use. This is accompanied by massive concerns about the materials safety upon contact with humans and organisms in the environment. To provide objective, evidence-based reporting on materials safety, scientists from different disciplines have joined forces to create a “Knowledge Base Materials.” Reliable and fact-based communication has a significant impact on various stakeholders and over the years, the communication tools have also been constantly updated.

In the early 2000s, a heated discussion arose about the need to analyse the risks of nanomaterials, alongside the opportunities they offer. Parallel to the 6^th^ and 7^th^ European Framework Program from 2003 to 2013 national research programs on nanotechnology were launched. Starting in 2006, the German Ministry for Education and Research (BMBF) promoted a series of laboratory research projects that laid the foundations for high-quality materials science and toxicological data and encouraged the communication of project results to various stakeholders with an interest in nanosafety. Subsequently, a consortium was mandated by the BMBF in 2009 to further develop these foundations and make them accessible to the public in a science communication project named DaNa (Data and Knowledge on Nanomaterials). The central intention of this initial information project was to provide an objective, fact-based presentation of nanomaterials and their safety. This was triggered by public concerns about the safety of nanomaterials at that time [e.g., (Prince sounds new nanotech alert, 2024)], and aimed at providing a sound scientific basis for public debate, which was sometimes rather driven by emotions than by scientific facts. A website and additional information materials like flyers or brochures were designed in German (www.nanopartikel.info) and English (www.nanoobjects.info) (and parts of it even in French) from the outset to achieve worldwide accessibility. The international availability of the DaNa information platform had a significant impact on both the number of visitors and the general visibility of the project.

In total, the BMBF funded from 2009 to 2023 a whole series of research projects dealing with the safety of various (nano)materials. The projects DaNa, DaNa2.0, and DaNa4.0 (note: there was no DaNa3) served as accompanying projects respectively, supporting project partners with networking as well as the dissemination of their research results via the DaNa website.

In addition to the central communication channel, the nanoobjects.info website, and information events for several stakeholders (e.g., pupils, students), the DaNa initiatives published their work (e.g., development of database, dissemination strategies, literature selection criteria) in specialised (Kimmig et al., 2013; Marquardt et al., 2013; Kühnel et al., 2014; Nau et al., 2016; Krug and Nau, 2017; Kühnel et al., 2017; Krug et al., 2018; Kuhnel et al., 2018; Nau et al., 2023) and general journals (Steinbach et al., 2012; Krug, 2014). Furthermore, a contact point was set up for citizens and consumers to discuss nanosafety issues directly with experts. Since the 2020s, the website has been expanded to include selected so-called advanced materials, in addition to various other measures (aspects of battery research, sustainability).

Over the years, the project website has successfully become a renowned information platform. This is shown by the fact that other initiatives have referred to DaNa, e.g., the EU NanoSafety Cluster (https://www.nanosafetycluster.eu) (Nanoriskgov, 2024; Nanosafetycluster, 2024), and the European Observatory for Nanomaterials (EUON) hosted by ECHA (https://euon.echa.europa.eu/safety) (EUON, 2024). Moreover, DaNa methods and work have received recognition at the EU level, e.g., in the NanoCommons User Guidance Handbook (https://nanocommons.github.io/user-handbook/) (Nanocommons, 2024).

In addition, also the access figures demonstrate growing interest from the public over the years. According to an internal project analysis (in line with data protection guidelines), in 2022 approx. 97,460 website visitors and 166,000 page views were recorded, demonstrating a need for information on the safety and application of various materials. This is also reflected in search engine rankings: the topics covered on nanopartikel.info and/or nanoobjects.info are in the top positions (Nau et al., 2024).

## 2 The team

Assessing the safety of nanomaterials is a complex process requiring the consideration of chemical-physiological material characterisation together with (eco)toxicological effects. For this, experts from various disciplines such as material sciences, chemistry, physics, biology, and toxicology teamed up to a powerful multidisciplinary team, which initially consisted of a core team from Empa and KIT for evaluation of human toxicology, UFZ for ecotoxicology, and DECHEMA and Fraunhofer IKTS for material properties of nanomaterials.

Over the years, various external experts from Germany, Austria, Switzerland, the United Kingdom, Denmark and Slovenia supported this core team. They were joined by IT specialists from KIT, who realized the technically sophisticated implementation of the knowledge database on nanoobjects.info as a unique selling point (see Table1).

**TABLE 1:** Team and external experts.

Temporary team members (2009–2023)	
Human toxicology	C. Marquardt (KIT, Germany)
	P. Wick, C. Hirsch, T. Bürki-Thurnherr (Empa, Switzerland)
	N. Bohmer (Empa, Switzerland/DECHEMA, Germany)
Ecotoxicology	A. Mattern, S. Reichelt, S. Wagner (UFZ, Leipzig, Germany)
Material information	V. Richter, S. Richter (Fraunhofer IKTS, Germany)
	B. Mathes, F. Paul, S. Espinoza, N. Möller (DECHEMA, Germany)
Web platform	C. Marquardt, D. Nehse, S. Kuhn (KIT, Germany)
IT	D. Kimmig, A. Schmidt (KIT, Germany)
Focus group analysis	M. Zschiesche (UfU, Berlin, Germany)
External experts (Affiliation at that time)	
Human toxicology	A. Duschl, M. Himly (University Salzburg, Austria)
	B. Rothen-Rutishauser, J. Caldwell (AMI, Switzerland)
Ecotoxicology	C. Tyler, R. Goodhead (University of Exeter, United Kingdom)
	B. Nowack, I. Hincapie (Empa, Switzerland)
	F. v.d. Kammer (University Vienna, Austria)
	N. Hartmann, A. Baun (Technical University of Denmark, DTU)
	A. Jemec Kokalj (University of Ljubljana, Slovenia)
Material information	J. Kreuter (Goethe University Frankfurt, Germany)
	A. Fink (AMI, Switzerland)
Exposure	T. Kuhlbusch (IUTA, Germany)
General consulting	S. Belluci (TA Swiss, Bern, Switzerland)
	M. Simko (Austrian Academy of Sciences, Vienna, Austria)
	C. Studer (BAG, Bern, Switzerland)

## 3 The challenges

For a well-balanced representation of knowledge on materials safety, we experienced various challenges, some of which will be briefly outlined here. For articles on human and environmental safety of advanced materials (including nanomaterials) only peer reviewed published data on (nano)materials was taken into account and underwent an internal project process. For this purpose, scientific literature for the respective compound or material was retrieved from common databases (e.g., PubMed, WoS, Scopus … ). As some (eco)toxicological testing of nanomaterials turned out to be prone to interferences (e.g., (Worle-Knirsch et al., 2006; Spohn et al., 2009), several misinterpretations (Priester J. et al., 2012; Ball, 2012; Priester J. H. et al., 2012; Gui et al., 2012; Lombi et al., 2012; Smith, 2012; Gui et al., 2015; DaNa-Project-Consortium, 2021a; SciTechDaily, 2021) and misconceptions (Yazdi et al., 2010; Schwab et al., 2011; Abdolahpur Monikh et al., 2021; DaNa-Project-Consortium, 2021b; DaNa-Project-Consortium, 2021c; Cross, 2021; Hou et al., 2021) of study results appeared in the press, most often in an alarming style.

A recent example is the controversial discussion about the biological effects of Titanium dioxide (TiO_2_) between the EU authorities on the one side and industry and several scientific experts on the other: In 2021, the European Chemicals Agency (ECHA) revised the classification of TiO_2_ based on high-dose tests on rats and results from questionable publications. Everyday products containing TiO_2_ (size-independent) must be labelled according to EUH212 as follows: “Warning! Hazardous respirable dust may be generated during use. Do not inhale dust” (ECHA, 2021a). Moreover, in 2021 the European Food Safety Agency (EFSA) re-evaluated studies from 2015 to 2020 and raised safety concerns as well (Younes et al., 2021; Boutillier et al., 2022). TiO_2_ (E171) was then banned as a food additive in the EU in August 2022. On 23 November 2022, the Court of Justice of the European Union (ECJ) annulled the Commission Delegation Regulation of 2019 as regards to the aforementioned classification and labelling of TiO_2_ in the CLP Regulation. In its judgment, the General Court ruled “that the Commission made a manifest error in its assessment of the reliability and acceptability of the study on which the classification was based, and incorrectly applied the classification criteria as laid down by the CLP Regulation to a substance that has the intrinsic property to cause cancer” (Union, 2022). International expert groups have doubts about the EFSA’s evaluation and are calling for this decision to be reconsidered in the EU (Driscoll, 2022; Kirkland et al., 2022).

Such topics confronted the DaNa researchers right from the beginning with the issue of reliability of studies and prompted us to develop a methodology for the selection of reliable studies as basis for our communication efforts. The central tool in this methodology became our quality criteria checklist (Nau et al., 2016; Krug et al., 2018; DaNa, 2021). This checklist compiles relevant criteria on basic particle characteristics, the study design, the biological test systems as well as the statistical evaluation of data. By defining both mandatory and voluntary criteria, it allowed us to select reliable studies, and only those were included into the knowledgebase that fulfilled minimum all mandatory criteria.

Further we were confronted with “data-rich” materials, such as nanosilver or nanoTiO_2_, for which unmanageable numbers of studies were found. Despite some efforts it was not possible to automatize the study quality evaluation. Hence, further selection criteria were included, and only studies published in journals with an impact factor of 2 or higher were considered. While using original publications for the articles on human and ecotoxicology, for the material information texts, also grey literature, as well as literature reviews were considered.

## 4 Communication

In general, scientists publish their results in scientific journals and report on their findings at conferences. The fact that scientists were reporting on nanotechnology, in particular on the safety of nanomaterials, via a web platform accessible to the general public, was really new in 2009. But focus group discussions in the forerunner project NanoCare and student focus groups in DaNa (Kuhlbusch et al., 2009) had shown that there was a need for reliable information on nanomaterials. The debate about possible risks should take place at an early stage of innovation and development with fact-based information and open communication in order to not repeat the mistakes of past debates, e.g., on green genetic engineering. Communicating science with different stakeholders is challenging, as the level of information and background knowledge differs individually. Discussing with other scientists is usually least challenging, as there is mostly a common basis, even though terminology may differ in detail. Stakeholders such as regulators are also grateful for reliable first-hand information. The main target group of the nanoobjects.info website is interested consumers. They often have questions about products containing nanotechnology or nanomaterials (Nano enabled products). Or they are concerned by advertisements and media reports and therefore consult the web platform for background information. It is therefore all the more important to provide reliable information on the safety of nanomaterials and writing articles in an understandable way. Open and understandable communication with the general public by scientists also demystifies the science and its often very specific terminology. For many consumers science is still practiced in ivory towers and there is no awareness on how science informs on safety issues. This is why scientists should communicate directly to the public. But that doesn’'t mean that every scientist has to do science communication. The acceptance of this work is still difficult, especially among scientific colleagues, because success in science is mainly measured in publications. It is not yet fully accepted that science communication requires resources and is not done on the side, as demonstrated by little funding options. The DaNa projects have taken on a pioneering role here, as they explicitly promoted work for the public. During the course of the projects, the team has continued to develop and the principles of science communication have become more and more integrated into the knowledge base. For example, by including formats such as a paper of the month, presenting scientific studies and highlighting the significance of their results for the general public.

Social media have played an increasingly important role in recent years. Channels on X (formerly Twitter), Mastodon and LinkedIn were established to reach an even wider audience. The social media channels complement the web platform, allowing the information to reach a younger target group as well.

## 5 The wishlist

From the outset, the DaNa experts intended to promote an objective, evidence-based discussion. This includes, in particular, the re-evaluation of published studies, as it is noticeable that many publications are not sufficiently qualified and/or often lack important, relevant information on the content, particularly with regard to toxicological statements. Some authors’ conclusions are often not comprehensible on the basis of the results presented (Song et al., 2009; Yazdi et al., 2010; Kolosnjaj-Tabi et al., 2015). Many publications in the area of nano(eco)toxicology lack important information on the physical-chemical characteristics of the particles, as well as on particle behaviour in testing media over time. This includes e.g., information on the size of internal structures, or the concentrations used in toxicity assays, so that the experiments are not reproducible. Therefore, a catalogue of relevant criteria was compiled by the DaNa team. It sets out clear requirements that the studies must meet in order to be included in our texts on materials and effects. Whether the criteria were fulfilled or not is carefully documented.

However, this will not be sufficient in the future, because with more than 80,000 published studies on the toxicological effects of nanomaterials, it is no longer possible to re-evaluate the results and content. Recent developments in AI point to an automated evaluation and review of studies. However, reliable quality evaluation is still essential, the more so for more complex advanced materials.

As shown by (Hristozov et al., 2012; Krug, 2014; Fernández-Cruz et al., 2018; Kirkland et al., 2022), only around 10%–30% of published studies on nanotoxicology can actually be used to assess a hazard or risk. The reason for this includes experimental design that may be not suitable for risk assessment purposes (e.g., use of only one test concentration), but also missing information on relevant parameters.

Missing information may be due to data that were not experimentally generated or data that exist but are simply not available due to several reasons. To overcome obstacles in data findability and re-use, a group of scientists formulated the FAIR principles (Wilkinson et al., 2016), standing for a concept to foster the Findability, Accessibility, Interoperability, and Reusability of scholarly data. The concept is now widely accepted and there are many efforts worldwide to implement it in practice (Mons et al., 2017), including the EU (European Open Science Cloud). There are activities in the field of material safety research as well (Jeliazkova et al., 2021). From this point of view, it would therefore also be desirable for each toxicological publication to include a supplement containing a form similar to a REACH dossier, in which all relevant (meta)data are listed in a machine-readable version (including the important properties of the material, the biological model, the treatment and the biological endpoints). This would ease the work of regulators, science communicators, and any re-users.

## 6 The future

Over the years of communicating about (nano)materials safety, also the material science and materials of interest have undergone tremendous changes. The materials became more complex in composition, nano and microscale particles were combined, and properties of materials were fine tuned for a specific application, leading to the field of advanced materials. Many novel developments for advanced materials are related to the huge societal transformation in front of us, to fight the triple planetary crisis of climate change, decline in biodiversity and pollution. Overall, in the EU, several strategies were brought on the way to support the transition to a more sustainable lifestyle (Green deal, Zero pollution action plan, Circular economy action plan). Here, advanced materials and material innovations are supposed to play a key role to e.g., make batteries for electromobility more efficient, improve the storage of energy from renewable sources, or provide efficient remediation methods to remove pollutants from the environment. But in addition, sustainability calls for improvements in material design (long-lived materials to prolong use phase of products) as well as in recyclability of materials from applications e.g., rare earth elements from batteries, to increase re-use of materials on the one hand, and reduce the environmental impacts of mining on the other hand. All this will lead to the constant development and market entry of novel materials, with new properties that may also affect materials impact on human and environmental health.

In recent years promising developments regarding the early detection of potential detrimental effects of novel materials have been made, e.g., the development of the Safe (and sustainable) by Design (SSbD) concept. Currently the concept is further developed to be put into practice by material developers (Caldeira et al., 2022). It must be avoided that such concepts fundamentally slow down research and innovation in general and thus prevent further scientific development. However, policy goals (such as EU Green deal (Commission, 2019), Chemicals Strategy for Sustainability (Commission, 2020)) need to be implemented into these concepts. In addition, the prediction of toxic effects of materials will further improve in the future, and this may also be useful for risk communication on novel materials (Oomen et al., 2015; Varsou et al., 2019; Braakhuis et al., 2021; Cross et al., 2024).

All these material innovations, being developed or applied in new combinations, support the need for constant, honest communication on potential hazardous effects for humans and the environment, based on facts and scientific evidence. In the EU, regulatory frameworks are constantly adopted to novel findings as evidenced by the implementation of nanomaterial-specific annexes and guidance into REACH, and adoption of cosmetics and pesticide regulations. It will be important also to communicate on regulatory issues to the society and inform on protective or restrictive measures taken by governments to manage potential risks of advanced materials. Currently, for example, there is a debate to include polymer materials into the registration required under REACH, and formulate criteria to identify materials of concern (ECHA, 2021b; European Commission and ECHA, 2021).

Further, digitalization (Krug, 2022) and the implementation of infrastructures for FAIR data (Dumit et al., 2023) hold promise to ease the DaNa teams future work by facilitating data search by e.g., data mining tools (also involving artificial intelligence) and in general allow access to toxicity data more easily.

## 7 Conclusion

Reflecting on the history of the DaNa projects and their achievement in building a unique knowledge base on materials safety accessible to everyone, there are promising developments in the field of toxicology, having a significant impact on our future work. To counteract fake news and “alternative facts,” transparent and evidence-based communication is crucial to build trust between stakeholders, including scientists, policymakers, regulatory authorities, and the public. Sustainable and environmentally friendly toxicology as applied in the concept of green toxicology, emphasizes the need to consider the environmental impact of chemicals and materials during the research and development process (Crawford et al., 2017). Ideally, chemicals harmful for humans and the environment will not enter the use phase, to avoid situations such as the current one with PFAS, which are extremely persistent and are now known as “forever chemicals.” They are suspected to cause cancer, and cause high economical burdens due to costs for healthcare, environmental monitoring, remediation and more [e.g., (Wee and Aris, 2023)]. Strengthening toxicology as an integral part of overall chemicals and materials research is essential for responsible and sustainable innovation.

Further, non-animal testing methods, often referred to as New Approach Methodologies (NAMs), play currently a pivotal role in toxicology. These alternatives aim to replace, reduce, or refine the use of animals in testing procedures by building a mechanistic understanding on effects of toxicants. This shift not only addresses ethical concerns related to animal welfare but also aligns with the broader goal of promoting environmentally friendly practices. NAMs include *in vitro* and high-throughput methods, computational models, and other innovative technologies that provide reliable and mechanistic data without relying on animal experiments.

Examples of long-term projects that communicate about materials safety and support green toxicology by publishing information on the web are “Nano-Trust” (https://www.oeaw.ac.at/ita/nanotrust/) and “DaNa” (www.nanoobjects.info, www.nanopartikel.info). These initiatives focus on understanding the potential risks associated with (nano)materials and provide reliable information for different stakeholders in a targeted manner. Both projects went beyond the typical funding period of three to 4 years, highlighting the need for sustained efforts and commitment to build trust and achieve meaningful communication activities.

In summary, the DaNa team took a long journey in science communication on (nano)materials safety. As with all things, standing still is not an option, and so constant adjustments have been made to current developments on the internet, in communication and also in material science and toxicology and we will continue to do so as the journey continues. Following the successful completion of the DaNa projects, the BMBF is funding safety and sustainability aspects of advanced materials as part of a new science communication project: MANTRA - data on innovative materials for sustainability and transfer. The new platform of the initiative www.materialneutral.info will be launched mid 2024.

## Data Availability

Publicly available datasets were analyzed in this study. This data can be found here: www.nanoobjects.info.
